# Characterization of Spermatogonial Stem Cells Lacking Intercellular Bridges and Genetic Replacement of a Mutation in Spermatogonial Stem Cells

**DOI:** 10.1371/journal.pone.0038914

**Published:** 2012-06-13

**Authors:** Naoki Iwamori, Tokuko Iwamori, Martin M. Matzuk

**Affiliations:** 1 Department of Pathology & Immunology, Baylor College of Medicine, Houston, Texas, United States of America; 2 Department of Molecular and Cellular Biology, Baylor College of Medicine, Houston, Texas, United States of America; 3 Department of Molecular and Human Genetics, Baylor College of Medicine, Houston, Texas, United States of America; 4 Department of Pharmacology, Baylor College of Medicine, Houston, Texas, United States of America; 5 Center for Reproductive Medicine, Baylor College of Medicine, Houston, Texas, United States of America; 6 Center for Drug Discovery, Baylor College of Medicine, Houston, Texas, United States of America; National Cancer Institute, United States of America

## Abstract

Stem cells have a potential of gene therapy for regenerative medicine. Among various stem cells, spermatogonial stem cells have a unique characteristic in which neighboring cells can be connected by intercellular bridges. However, the roles of intercellular bridges for stem cell self-renewal, differentiation, and proliferation remain to be elucidated. Here, we show not only the characteristics of testis-expressed gene 14 (TEX14) null spermatogonial stem cells lacking intercellular bridges but also a trial application of genetic correction of a mutation in spermatogonial stem cells as a model for future gene therapy. In TEX14 null testes, some genes important for undifferentiated spermatogonia as well as some differentiation-related genes were activated. TEX14 null spermatogonial stem cells, surprisingly, could form chain-like structures even though they do not form stable intercellular bridges. TEX14 null spermatogonial stem cells in culture possessed both characteristics of undifferentiated and differentiated spermatogonia. Long-term culture of TEX14 null spermatogonial stem cells could not be established likely secondary to up-regulation of CDK4 inhibitors and down-regulation of cyclin E. These results suggest that intercellular bridges are essential for both maintenance of spermatogonial stem cells and their proliferation. Lastly, a mutation in *Tex14^+/−^* spermatogonial stem cells was successfully replaced by homologous recombination *in vitro*. Our study provides a therapeutic potential of spermatogonial stem cells for reproductive medicine if they can be cultured long-term.

## Introduction

Stem cells are defined as cells that possess both self-renewal activity and differentiation potential. Because of these characteristics, stem cells are expected to be therapeutic targets for regenerative medicine and can be used in the future for the correction of genetic defects [1], [2]. Once the mutation is repaired, either the stem cells or differentiated cells could be transferred to the individual to reconstitute or correct the damaged cells or organs. At present, there have been several reported trials utilizing stem cells as a means of regenerative therapy[3]–[5]. One problem for the utilization of stem cells for regenerative medicine is the difficulty of culture and expansion of stem cells *in vitro*. Indeed, stem cells used in the regenerative therapies described above are embryonic stem cells (ESCs) and induced pluripotent stem cells (iPSCs), for which efficient culture systems have been established[3]–[5]. Among the various stem cells, spermatogonial stem cells (SSCs) are the only stem cells derived from an adult animal and for which both long-term culture systems and transplantation techniques have been developed [6]–[9].

SSCs arise from gonocytes during postnatal development of the testis, possess self-renewal potential, and allow spermatogenesis to continue throughout adult life [10], [11]. SSCs can be isolated from both postnatal and adult testes and be maintained long-term *in vitro* in an undifferentiated state in the presence of glial cell line derived neurotrophic factor (GDNF) [7]–[9]. SSCs can reconstitute spermatogenesis when they are transplanted into germ cell-depleted testes [6]. A number of genes, including cell surface markers, transcription factors, and other proteins, have been identified as markers of undifferentiated spermatogonia, and some of these proteins are essential for self-renewal of SSC, although the molecular mechanisms regulating SSCs still remain elusive [12]–[27].

During spermatogenesis, a unique structure called the intercellular bridge is observed [28], [29]. As germ cells begin the process of differentiation, they are connected with neighboring sister germ cells through the intercellular bridge. We previously identified testis-expressed gene 14 (TEX14) as an essential component in the intercellular bridges during spermatogenesis [30]–[32]. When we produced a knockout of TEX14, spermatogenesis was arrested and only spermatogonia remained even in 1-year-old testes [30]. Moreover, most PLZF-positive spermatogonia existed as single cells in TEX14 null testes, suggesting that TEX14 null SSCs were at least maintained even in aged testes. However, whether SSCs without intercellular bridge can self-renew, differentiate, and proliferate remains unclear.

Herein, we analyzed the characteristics of testes without intercellular bridges by examining the expression of SSC marker genes, established long-term cultures of SSC with a mutation in *Tex14*, and showed that we could correct a genetic defect in *Tex14^+/−^* spermatogonia by homologous recombination to reveal the therapeutic potential of SSCs for regenerative medicine.

**Table 1:** Primer sequences used in the study.

Gene name	Forward primer	Reverse primer
Used in qPCR		
Cd9	TGCATGCTGGGATTGTTCTTC	GGCGGCGGCTATCTCAA
Cdh1	ACCGATTCAAGAAGCTGGC	ACCATCCTAACACAGACAGTCC
Epcam	TGCTCCAAACTGGCGTCTAA	TCCCAGACTTGCTGTGAGTCA
Gfra1	TACCACCAGCATGTCCAATGAA	GTAGCTGTGCTTGGCTGGAACT
Ret	GGCTGTCCCGAGATGTTTATG	GACTCAATTGCCATCCACTTGA
Zbtb16	CACACTCAAGAGCCACAAGC	ATCATGGCCGAGTAGTCTCG
Bcl6b	CGCCAGGAAGTGAGTTTTTCA	GCTCCAGCCCCGATGAG
Id4	GAGACTCACCCTGCTTTGCT	ATGCTGTCACCCTGCTTGTT
Nanos2	AACTTCTGCAAGCACAATGG	CCGAGAAGTCATCACCAG
Taf4b	AGCCTAACAGCCACCAAACC	TGAATTCTCAGCGGCATG
Neurog3	GCCTCATTGGAGGAATTCC	AGATGCTTGAGAGCCTCCAC
Stra8	ACAAGAGTGAGGCCCAGCAT	CCTCTGGATTTTCTGAGTTGCA
Kit	GCCACGTCTCAGCCATCTG	GTCGGGATCAATGCACGTCA
Ddx4	AGGACGAGATTTGATGGCTTGT	GGCAAGAGAAAAGCTGCAGTCT
Tex14	AAATAGTAGGAGTATGGCGTCTG	CCATTTCAAGTGTGCCTCTC
Ink4a	GTGTGCATGACGTGCGGG	GCAGTTCGAATCTGCACCGTAG
Arf	GCTCTGGCTTTCGTGAACATG	TCGAATCTGCACCGTAGTTGAG
Cdkn1a	GCAGATCCACAGCGATATCC	CAACTGCTCACTGTCCACGG
Cdkn1b	AAGGGCCAACAGAACAGAAG	GGATGTCCATTCAATGGAGTC
Clnd1	GTTCATTTCCAACCCACCC	CTCAGATGTCCACATCTCGC
Clnd2	GATCACCCACACTGATGTGG	ATGACGAACACGCCTCTCTC
Clnd3	ATGTCACAGCCATTCACCTG	CTGGTTGAGTGGGAAGGAAG
Clne	CTGGTTGAGTGGGAAGGAAG	TAGAGCACAGCATCTGCAGG
Used in genotyping		
Tex14 F1	GACGGTACTCCTGCTCTTGG	
Tex14 F2	GTACCTTCTGAGGCGGAAAG	
Tex14 F3	GCAGGGTCATGTCATGTAGG	
Tex14 R1	GACAGCCAGGGGTTACAGAG	
Tex14 R2	GGGTGGTTCAGAGCCATCTA	
Tex14 R3	GGGGAACTTCCTGACTAGGG	
Tex14 RN	ATTCGCAGCGCATCGCCTTCTATCGCC	

## Materials and Methods

### Animals and Cell culture

TEX14 heterozygous mice [30], which were C57BL/6J: 129S5/SvEvBrd hybrid background, were crossed to actin-GFP transgenic mice. *Tex14^+/−^* and GFP double-mutant mice were backcrossed to DBA strain for at least two generations before they were intercrossed. All mouse experiments were performed in accordance with protocols approved by the Institutional Care and Use Committee of Baylor College of Medicine.

**Figure 1:**
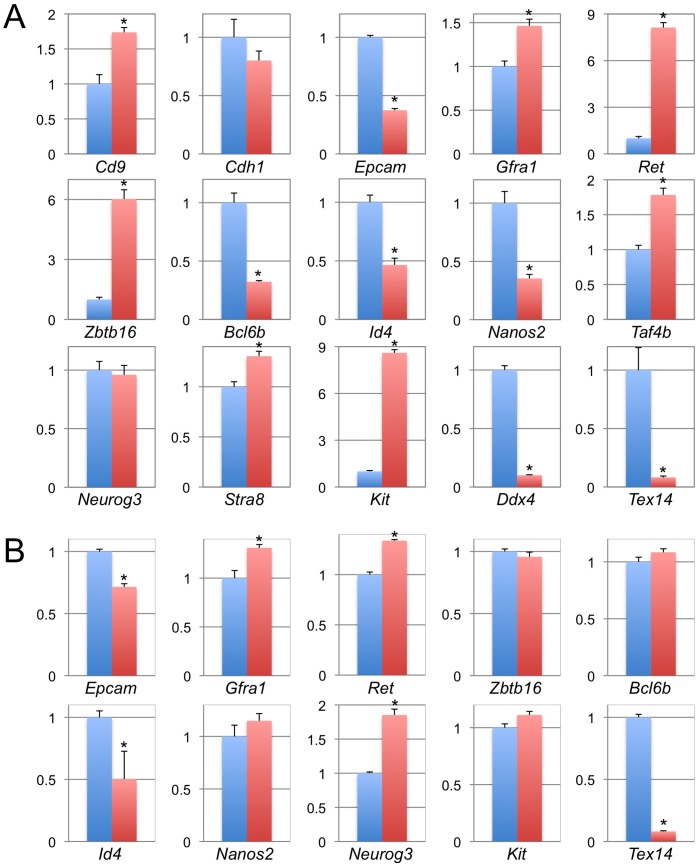
Expression profiles of SSC markers in *Tex14^−/−^* testis and spermatogonia. Relative expression patterns of the indicated genes in *Tex14^+/−^* (blue) and *Tex14^−/−^* (red) testis at 8 weeks of age (A) and in CD9-positive *Tex14^+/−^* (blue) and *Tex14^−/−^* (red) spermatogonia (B) were quantitatively analyzed. All of the expression levels were normalized to *Gapdh* expression. * indicates that there is significant difference (P<0.05).

Establishment of spermatogonial stem cell lines was performed as previously described [7]. Briefly, testes and tails were collected from GFP-positive pups at postnatal 7 days and processed for establishment of spermatogonial stem cell lines and genotyping, respectively. After removal of the tunica, isolated testes were treated with a two-step enzymatic digestion with collagenase and trypsin [33]. Testicular cells were suspended in SSC media supplemented with 10 ng/ml rat glial cell derived neurotrophic factor (GDNF; R&D systems), 20 ng/ml mouse epidermal growth factor (EGF; eBiosciences), 10 ng/ml human basic fibroblast growth factor (bFGF; eBiosciences), and 10^3^ U/ml mouse leukemia inhibitory factor (LIF; Millipore) and seeded onto gelatinized dish. After seven days of culture, SSCs were collected by gentle pipetting and seeded onto inactivated mouse embryonic fibroblast (MEF) feeder layers. SSCs were cultured for at least one month before use in additional experiments.

### Magnetic Activated Cell Sorting

Testicular cells prepared as described above were incubated with rat anti-CD9 antibodies (BD Biosciences) on ice for 30 min, followed with anti rat IgG microbeads (Miltenyi Biotec) on ice for 20 min. CD9-positive cells were separated by MACS cell separation system (Miltenyi Biotec) according to the manufacturer’s instructions.

**Figure 2:**
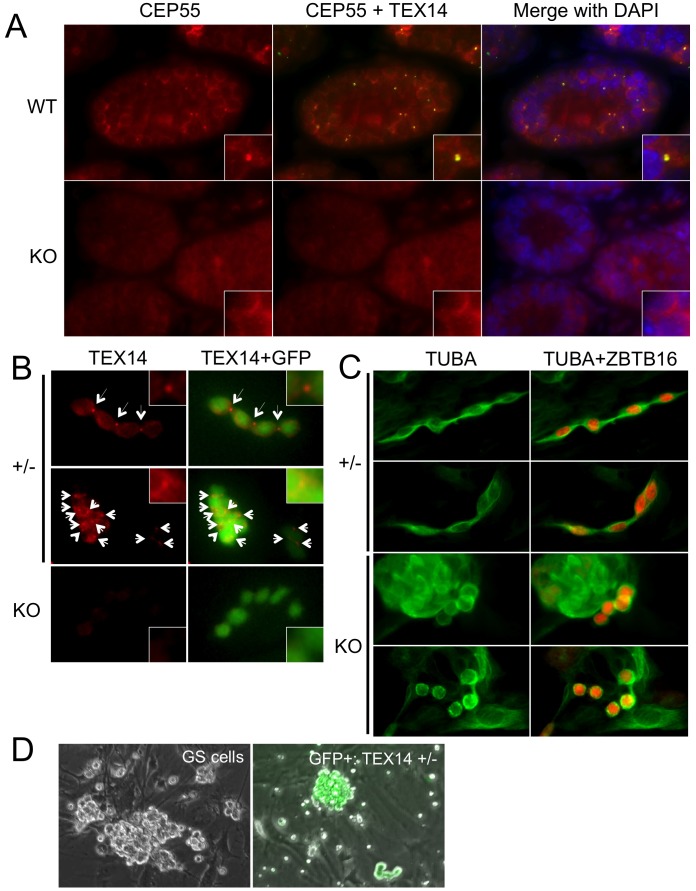
Detection of intercellular bridge formation and establishment of spermatogonial stem cells in culture. A. Detection of intercellular bridge proteins in *Tex14^+/−^* and *Tex14^−/−^* testis. Immunofluorescence images of CEP55 (red) and TEX14 (green) and nucleus (DAPI) in 8-week old *Tex14^+/−^* (+/−) and *Tex14^−/−^* (KO) testis are shown. Insets indicate higher magnification images. B. Detection of intercellular bridges in short term culture of *Tex14^+/−^* and *Tex14^−/−^* spermatogonia. GFP (green) positive spermatognia were cultured and stained with TEX14 antibody (red) at second passage. Arrows indicate TEX14 positive intercellular bridges. C. Cytoskelton of short-term culture of *Tex14^+/−^* and *Tex14^−/−^* spermatogonia. Immunofluorescence images of TUBA (alpha-tubulin: green) and ZBTB16 (red) in *Tex14^+/−^* (+/−) and *Tex14^−/−^* (KO) spermatogonia are shown. D. Morphology of established spermatogonial stem cells in culture. Typical morphologies of cultured wildtype and *Tex14^+/−^* SSCs at 3 months of culture are shown.

**Figure 3:**
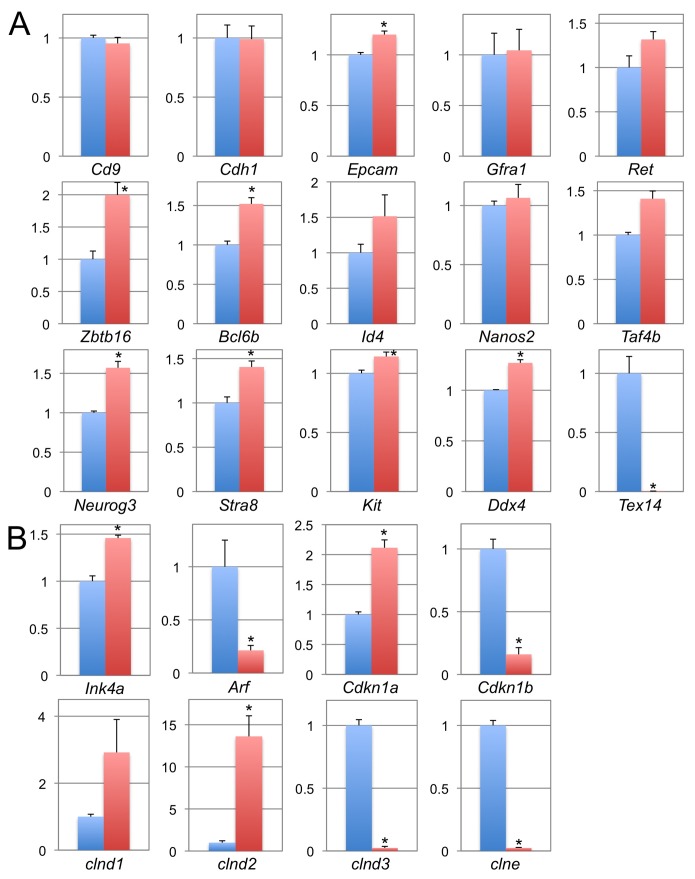
Expression profiles of SSC markers and cell cycle regulators in *Tex14^+/−^* and *Tex14^−/−^* SSCs. Relative expressions of SSC markers (A) and cell cycle regulators (B) in *Tex14^+/−^* (blue) and *Tex14^−/−^* (red) SSCs were quantitatively analyzed. All of the expression levels were normalized to *Gapdh* expression. * indicates that there is significant difference (P<0.05).

### Quantitative RT-PCR

Total RNA from testes and SSCs were extracted using the RNA mini and micro kit (QIAGEN), respectively, according to the manufacturer’s instructions and reverse transcribed using Superscript III reverse transcriptase (Invitrogen) and an oligo-dT primer (Invitrogen). PCR amplifications were performed using SYBR Green PCR Master Mix (ABI) and analyzed by the ABI 7500 sequence detection system. The primers used in the experiments were shown in Table 1.

### Immunofluorescence

For visualization of intercellular bridges, cells were fixed with 2% paraformaldehyde for 10 min and permeabilized with 0.5% NP-40 for 15 min. After incubation with goat anti-TEX14 antibodies [30] at 4°C overnight, intercellular bridges were visualized by Alexa 594 conjugated anti-goat IgG (Invitrogen). For immunofluorescence of SSCs in culture, fixed and permeabilized cells were incubated with primary antibodies at 4°C overnight, followed by Alexa 488 and Alexa 564 conjugated secondary antibodies (Invitrogen). Following primary antibodies were used; rabbit anti-ZBTB16 (PLZF; Santa Cruz Biotechnology), mouse anti-ZBTB16 (PLZF; Calbiochem), mouse anti-TUBA (alpha-tubulin; Santa Cruz Biotechnology), rabbit anti-ARF (Abcam), mouse anti-INK4A (p16; Santa Cruz Biotechnology), mouse anti-CDKN1A (p21; Santa Cruz Biotech.), and mouse anti-CCNE (cyclin E; Santa Cruz Biotech.) antibodies. For immunofluorescence of tissue sections, paraformaldehyde fixed sections were retrieved by microwave, and then incubated with goat anti-TEX14 [30] and guinea pig anti-CEP55 [32] antibodies overnight at 4°C, followed by Alexa 488 and Alexa 594 conjugated secondary antibodies (Invitrogen) for 1 hour at room temperature. Fluorescent sections were mounted with VECTASHIELD containing DAPI (VECTOR Laboratories).

### Generation of a Genetic Rescue Construct and Homologous Recombination in SSCs

A rescue construct was generated as follows: 8.3 kb of the 129S7/SvEvBrd-Hprtb-m2 genomic region containing exons 9 and 10 of the mouse *Tex14* gene [34] was inserted into pBluescript SK containing diphtheria toxin A for negative selection (pDTA.3 kindly provided by Dr. Pumin Zhang, Baylor College of Medicine) and a *loxP*-flanked *Pgk1-Neo* cassette was inserted 2 kb downstream of exon 10 of *Tex14*. The linearized rescue construct was electroporated into *Tex14^+/−^* SSCs. SSC clones were selected in SSC media supplemented with G418 and 6-thioguanine. Targeted clones were screened by genomic PCR using primers shown in Table 1.

**Figure 4:**
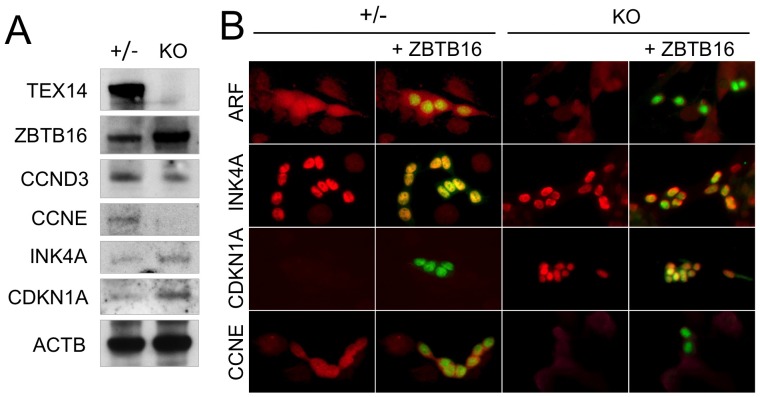
Alterations of cell cycle regulators in spermatogonial stem cells by lack of intercellular bridges. Protein expression of cell cycle regulators in *Tex14^+/−^* and *Tex14^−/−^* SSCs were analyzed by immunoblot (A) and immunostaining (B). Immunofluorescence images (B) of indicated cell cycle regulators (green) and ZBTB16 (red) in *Tex14^+/−^* (+/−) and *Tex14^−/−^* (KO) SSCs are shown.

**Figure 5:**
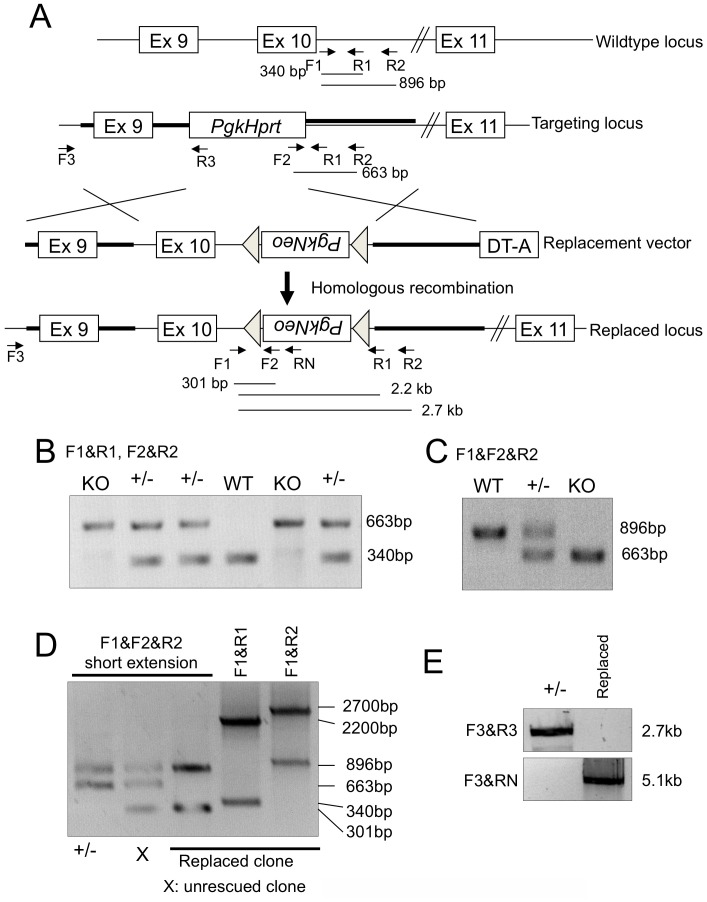
Genotyping of the rescued *Tex14* allele. A. Strategy of gene replacement of the *Tex14* mutation. The *Pgk-Hprt*, which was inserted into exon 10 of the *Tex14* gene, was replaced with exon 10 of *Tex14* and a *loxP* flanked *Pgk-Neo* cassette. Locations of primers used for genotyping and predicted size of the PCR products are shown. B and C. Genotyping of the *Tex14* mutation. Mutation of the *Tex14* gene was confirmed by genomic PCR using primer pairs F1 and R1, F2 and R2 (B), and F1 and F2 and R2 (C). D. Genotyping of the replaced allele of *Tex14* in *Tex14^+/−^* SSCs. Genomic PCR using indicated primer pairs and conditions are shown. Lane 1: *Tex14^+/−^* SSCs, Lane 2: unreplaced SSC clone, Lanes 3–5: replaced SSC clone. E. Long range genomic PCR to confirm homologous recombination. Genomic PCR using indicated primer pairs are shown.

## Results

### Molecular Analysis of SSC Markers in Tex14 Null Testes

Expression patterns of genes, known to be transcriptionally active in spermatogonial stem cells, were analyzed in TEX14 null testes, because characteristics of *Tex14^−/−^* spermatogonial stem cells, such as self-renewal activity, differentiation potential, and proliferative activity, remained elusive. For the expression of cell surface markers of spermatogonial stem cells, *Cd9, Gfra1,* and *Ret* were significantly increased in *Tex14^−/−^* testes, although *Epcam* was decreased (Fig. 1A). Up-regulation of *Gfra1* and *Ret* suggests enrichment of undifferentiated spermatogonia in *Tex14^−/−^* testes, since *Gfra1* and *Ret* are receptors for GDNF, which is an essential growth factor for SSCs. For the expression of intercellular markers of spermatogonial stem cells, *Zbtb16* (also called *Plzf*), *Stra8*, and *Taf4b* were increased in *Tex14^−/−^* testes, whereas *Bcl6b*, *Id4*, and *Nanos2* were decreased in *Tex14^−/−^* testes (Fig. 1A). Enhanced expression of the three former genes suggests that undifferentiated spermatogonia might be enriched in *Tex14^−/−^* testes. Suppressed expression of the latter three genes, however, suggests that the ratio of undifferentiated spermatogonia to differentiated spermatogonia remaining in *Tex14^−/−^* testes might not be very high, or that spermatogonia remaining *Tex14^−/−^* testes might have both differentiated and undifferentiated characteristics even if they were existed as single spermatogonia. Expression of *Ddx4* (also known as *Mvh*) was extensively lower in *Tex14^+/−^* testes, probably because *Ddx4* is expressed not only in spermatogonia but also in later stage germ cells, and there were only spermatogonia enriched in *Tex14^−/−^* testes (Fig. 1A).

Next, spermatogonia were enriched from whole testis using CD9 as a marker of undifferentiated spermatogonia. When expression of cell surface markers was analyzed, *Epcam* was significantly lower in *Tex14^−/−^* spermatogonia, and *Gfra1* and *Ret* were significantly higher (Fig. 1B). However, differences of expression of those genes between *Tex14^+/−^* and *Tex14^−/−^* spermatogonia were decreased. For the expression of intracellular markers, expression of *Zbtb16, Bcl6b,* and *Nanos2* were not significantly changed between *Tex14^+/−^* and *Tex14^−/−^* spermatogonia (Fig. 1B). Although *Id4* expression was significantly lower in *Tex14^−/−^* spermatogonia, the difference between *Tex14^+/−^* and *Tex14^−/−^* spermatogonia was decreased (Fig. 1B). When differentiation related markers were analyzed, *Neurog3* expression was higher in *Tex14^−/−^* spermatogonia, but *Kit* expression in *Tex14^−/−^* spermatogonia was as high as *Tex14^+/−^* spermatogonia (Fig. 1B). These results for the expression profiles in enriched spermatogonia suggest that spermatogonia lacking intercellular bridge might not be very different from normal spermatogonia and that CD9-positive *Tex14^−/−^* spermatogonia might have both undifferentiated and differentiated characteristics.

### Morphological Analysis of Tex14 Heterozygous and Homozygous Mutant SSCs in Culture

The disappearance of intercellular bridges in *Tex14^−/−^* testes was previously confirmed by immunostaining of TEX14 and CEP55, both of which are essential components for intercellular bridges to be stabilized [32]. There is no clear localization of both TEX14 and CEP55 in *Tex14^−/−^* testes, indicating that no stable intercellular bridges form in *Tex14^−/−^* testes (Fig. 2A).

Before establishment of long-term cultures of spermatogonial stem cells, characteristics of spermatogonia without intercellular bridges were analyzed in short-term cultures of *Tex14^−/−^* spermatogonia. Surprisingly, *Tex14^−/−^* spermatogonia could form chain-like structure, even though no intercellular bridges formed (Fig. 2B). *Tex14^−/−^* spermatogonia could also form cluster-type colonies, although the size of these clusters was small and the number of cells in these clusters was less than ten (data not shown). When intercellular bridges were visualized in isolated *Tex14^+/−^* and *Tex14^−/−^* spermatogonia using TEX14 staining, clear TEX14-positive intercellular bridges were detected in both chains and clusters of *Tex14^+/−^* colonies but not in *Tex14^−/−^* colonies (Fig. 2B). To confirm lack of intercellular bridges in *Tex14^−/−^* spermatogonia, alpha-tubulin (TUBA) in cultured spermatogonia was determined. TUBA expression in *Tex14^+/−^* spermatogonia was observed to be stretched and showed that cells were connected with neighboring cells, whereas TUBA expression in *Tex14^−/−^* spermatogonia was round shaped and showed that each cell was not connected with neighboring cells (Fig. 2C). These results indicate that *Tex14^−/−^* spermatogonia definitely lack intercellular bridges although they could form small clusters and chain-like structures without interconnections to neighboring cells.

### Molecular Analysis of Tex14 Heterozygous and Homozygous Mutant SSCs in Culture

Gene expression of several genes, which are essential for spermatogonial stem cells, was examined in short-term cultures of spermatogonia. A number of stem cell related genes (*Epcam*, *Ret*, *Zbtb16*, *Bcl6b*, *Taf4b*, *Neurog3*, and *Stra8*) were significantly upregulated in *Tex14^−/−^* spermatogonia, while differentiation related genes (*Kit* and *Ddx4*) were simultaneously upregulated (Fig. 3A). The expression of *Id4* and *Nanos2*, whose expression are restricted to single cells and clusters with less than four aligned spermatogonia, respectively, were not significantly changed in *Tex14^−/−^* spermatogonia, even though single spermatogonia were enriched in *Tex14^−/−^* intercellular bridge less spermatogonia (Fig. 3A). Upregulation of *Neurog3* and *Stra8* was also intriguing, because these genes are known to be expressed at a higher level in differentiating spermatogonia rather than undifferentiated spermatogonia [17], [25], [35]. These results suggest that *Tex14^−/−^* spermatogonia are already committed to differentiation, even though they possessed characteristics of undifferentiated spermatogonia.

We also analyzed the expression of cell cycle related genes in *Tex14^−/−^* spermatogonia. While expression of CDK4 inhibitors, *p16 Ink4a* and *Cdkn1a*, are increased, *p19 Arf* and *Cdkn1b* were decreased in *Tex14^−/−^* spermatogonia (Fig. 3B). When expression levels of cyclins were analyzed, *cyclin D1* and *cyclin D2* were significantly upregulated, and *cyclin D3* and *cyclin E* were not detected in *Tex14^−/−^* spermatogonia (Fig. 3B). Down-regulation of p19 ARF, cyclin D3, and cyclin E and up-regulation of p16 INK4A and CDKN1A were confirmed at protein level (Fig. 4). Notably, CDKN1A in *Tex14^+/−^* spermatogonia was undetectable, whereas CDKN1A in *Tex14^−/−^* spermatogonia was high, suggesting that lack of intercellular bridges inhibit cell proliferation by induction of CDKN1A. Down-regulation of cyclin E could be induced by inhibition of CDK4 activity. These results suggest that cell cycle progression of *Tex14^−/−^* spermatogonia was inhibited by two different CDK4 inhibitors even though CDK4 activity in *Tex14^−/−^* spermatogonia could be activated by expression of its related cyclins.

### Establishment of Tex14 Heterozygous and Homozygous Mutant SSCs in Culture

We next attempted to establish long-term cultures of *Tex14^−/−^* and *Tex14^+/−^* spermatogonial stem cells. We successfully established long-term cultures of spermatogonial stem cell from wild-type and GFP-positive *Tex14^+/−^* mice (Fig. 2D). *Tex14^+/−^* spermatogonial stem cells showed similar colony morphology as wild-type cells (Fig. 2D). However, *Tex14^−/−^* spermatogonia could not be maintained for long-term, although they could form colonies and grow for short-term as shown above.

### Replacement of the Genetic Defect in Tex14 Heterozygous Mutant SSCs in Culture

To replace the genetic defect in *Tex14^+/−^* spermatogonial stem cells in which exon 10 was replaced with a *Pgk1-Hprt* cassette in one allele of genome, a replacement construct to “knockin” exon 10 into the deleted genome was generated (Fig. 5A). In this replacement construct, the *Pgk1-Neo* cassette was flanked with loxP sequences so that it could be removed after insertion into the genome. After transfection of this replacement construct into *Tex14^+/−^* spermatogonial stem cells, the stem cells were expanded and genotyped using genomic PCR. Seventeen colonies could be expanded and one colony was confirmed to have correct replacement of the deleted exon 10 of *Tex14* in the genome (Fig. 5). Although these genetically replaced spermatogonial stem cells were transplanted into germ cell-depleted testes, unfortunately no pups were obtained.

## Discussion

In the present study, characteristics of spermatogonia without intercellular bridges were analyzed. By gene expression profiling, *Tex14^−/−^* spermatogonia possessed not only characteristics of undifferentiated spermatogonia but also characteristics of differentiated spermatogonia. To conclude if spermatogonial stem cells without intercellular bridges could self-renew or not, further experiments using transplantation technique could be required. However, it is likely impossible to evaluate self-renewal activity of *Tex14^−/−^* SSCs by detection of reconstituted colonies after transplantation because our results suggest that proliferative activity of *Tex14^−/−^* SSCs is low and *Tex14^−/−^* SSCs may only form small colonies. At least *in vivo*, *Tex14^−/−^* SSC are able to self renew, since there are a number of spermatogonia even in one year-old *Tex14^−/−^* testes [30].

In our study, one surprising finding is the formation of chain-like colonies by *Tex14^−/−^* SSCs, although our previous reports showed that most spermatogonia in *Tex14^−/−^* testes lack chain formation and exist as a single state but not in paired and aligned forms as observed in wild-type adult testes [30]. In addition, we confirmed *Tex14^−/−^* spermatogonia were not connected with neighboring cells, even if they form chain-like structure. This suggests that there may be adhesion proteins expressed on the surface of the dividing SSCs that allow them to adhere to one another in a chain-like structure. Alternatively, the chain-like structure may be beneficial for survival and/or cell division of the SSCs because of paracrine growth factors secreted from neighboring SSCs. There may also be some differences in the characteristics between spermatogonial stem cells *in vivo* and *in vitro*. All postnatal male germ cells are surrounded by Sertoli cells *in vivo,* whereas cultured SSCs are free to move and to divide and their proliferation could be accelerated *in vitro*. As we previously showed, TEX14 functions as a stabilizer of intercellular bridge to avoid cell abscission by interaction with and inhibition of CEP55 function [32]. Midbody formation occurs during cellular division even in *Tex14^−/−^* spermatogonia. As shown in Figure 2A, there was no localization of CEP55 to midbodies in 8-day old testes where spermatogonia actively proliferate. These results suggest that proteins required for cell abscission might not be expressed at very high levels in the *Tex14^−/−^* midbody, and this midbody might act as a transient intercellular bridge. Alternatively, intercellular bridge might regulate the movement of spermatogonia, because previous reports showed that chains of spermatogonia extensively move around in seminiferous tubules [36], [37]. *Tex14^−/−^* spermatogonia might not be able to move actively after cell division because of a lack of intercellular bridges.

*Tex14^−/−^* spermatogonia display both activation and repression of proliferative activity according to our expression profiling. *Tex14^−/−^* spermatogonia can proliferate even though expression of CDK inhibitors were activated and cyclin E expression was repressed, because some chains of *Tex14^−/−^* spermatogonia were detected in the culture *in vitro*. However, there must be a reduced rate of proliferation since the *Tex14^−/−^* colonies were smaller in short-term culture, and *Tex14^−/−^* SSCs could not be expanded in long-term culture. Otherwise, *Tex14^−/−^* SSCs could proliferate in their first few divisions and might subsequently enter cellular senescence. Since down-regulation of cyclin E expression could be affected by up-regulation of CDK inhibitors, especially CDKN1A, up-regulation of CDKN1A may be critical for inhibition of cellular proliferation of *Tex14^−/−^* spermatogonia. As shown in Figure 2C, cytoskelton of *Tex14^−/−^* spermatogonia were dramatically changed by lack of intercellular bridges. Checkpoint mechanisms monitoring cellular cytoskelton might induce up-regulation of CDKN1A. Formation of intercellular bridges, at least, could be important for continuous proliferation of spermatogonia.

An attempted replacement of a genetic defect in spermatogonial stem cells was also presented in this study. This is the first report showing a genetic correction *in vitro* using spermatogonial stem cells, although there are reports of homologous recombination in spermatogonial stem cells [38], [39]. Unexpectedly, *Tex14^−/−^* spermatogonial stem cells could not be expanded, although there are many spermatogonia in *Tex14^−/−^* testes. Therefore, we used *Tex14^+/−^* spermatogonial stem cells as a model of genetic correction. If spermatogonial stem cells could be easily expanded from infertile animals or patients, this methodology could be applicable. Of course, there are some ethical issues that need to be considered if this technique is used in the therapy of infertile patients since this method could produce genetically engineered offspring. Therefore, at this time, this technique is appropriate for therapy of infertile animals except for human. If rare animals are infertile or subfertile because of genetic defects, this methodology would be very powerful to solve this problem.
